# LOVE AMONG OLDER AFRICAN AMERICAN COUPLES: AN ACTOR PARTNER INTERDEPENDENCE MODEL ANALYSIS

**DOI:** 10.1093/geroni/igac059.3056

**Published:** 2022-12-20

**Authors:** Kadija Mussa, Chalandra Bryant

**Affiliations:** University of Minnestoa, Falcon Heights, Minnesota, United States; University of Minnesota, Minneapolis, Minnesota, United States

## Abstract

Older women are often described as being asexual and uninterested in sex or intimacy (McHugh & Interligi, 2015). Thus, most research examining older couples describes those couples as primarily enjoying companionate or compassionate love – a type of love reflecting care and concern for another person (Allen et al., 2018). Unlike companionate or compassionate love, passionate love refers to a “state of intense longing for union with another” (Hatfield & Rapson, 1993, p. 67). Relatively little is known about passionate love and older couples (Hatfield & Rapson, 1993); moreover, far less is known about passionate love among African American older couples. Using data collected from African American couples (332 couples aged 20 to 39 and 90 couples aged 40 to 79), Actor-Partner Interdependence Models were used. For both age groups, 20 to 39 and 40 to 79, husbands’ and wives’ reports of marital quality were significantly associated with each other at Time 1. Husbands’ and wives’ reports of passionate love (assessed at Time 2) were not significantly associated with each other – for either age group under study. Cross paths (partner effects) were not significant for either of the two age groups; wives’ marital quality (Time 1) did not significantly predict husbands’ passionate love (Time 2), nor did husbands’ marital quality (Time 1) predict wives passionate love (Time 2). It is important to note that the older and younger age groups exhibited a similar pattern of results, suggesting that passion and physical intimacy may operate in similar ways for both.

